# Supplementary material for: Building of an Internal Transcribed Spacer (ITS) Gene Dataset to Support the Italian Health Service in Mushroom Identification
Source: Foods. 2021 May 25;10(6):1193. doi: 10.3390/foods10061193 (PMC8227961; doi:10.3390/foods10061193)
Supplement: Supplementary file 1 [file foods-10-01193-s001.zip › foods-1200583-supplementary.pdf]

Table S1. Target species of the ITS gene dataset with relative edibility. The number of retrieved ITS sequences in GenBank (G) and BOLD is reported, with the number of filtered ITS sequences maintained by the systematic double-step filtering process. The intra-species overall mean distance is also reported. E: Edible; SNE: Suspected not edible; NE: Not edible; T: Toxic; M: Mortal

							Retrieved ITS sequences (n)			Double-step filtering (n)		
					**Species**	**edibility**	**GB**	**BOLD**	**GB+BOLD**	**1st**	**2nd**	**Overall mean dstance**
Basiomycota	Agaricomycetes	Agaricales	Agaricaceae	Agaricus	*A. arvensis*	E	33	1	34	32	29	0.02
					A. bitorquis	E	61	2	63	50	50	0.01
					A. bresadolanus	T	6	0	6	6	6	0.03
					A. iodosmus	T	8	0	8	8	8	0
					A. menieri	T	4	0	4	4	4	0
					A. moelleri	T	16	3	19	15	14	0.02
					A. phaeolepidotus	T	6	0	6	6	6	0
					A. pseudopratensis	T	9	0	9	9	7	0
					A. sylvicola	E	5	0	5	4	4	0
					A. xanthodermus	T	33	6	39	35	28	0.01
				Chlorophyllum	*C. brunneum*	SNE	23	3	26	25	25	0
					C. rhacodes	SNE	19	0	19	15	14	0.06
				Lepiota	*L. brunneoincarnata*	M	20	0	20	20	19	0.01
					L. clypeolaria	T	39	3	42	31	27	0.02
					L. cristata	T	91	4	95	78	78	0.03
					L. erminea	T	3	3	6	3	2	0.01
					L. helveola	M	2	0	2	1	1	-
					L. ignivolvata	T	3	0	3	3	3	0.01
					L. magnispora	T	33	3	36	33	33	0.06

					L. subincarnata	M	23	0	23	23	22	0
				Leucoagaricus	*L. americanus*	T	10	1	11	9	9	0.03
					L. leucothites	T	38	2	40	37	37	0.02
				Leucocoprinus	*L. cepistipes*	SNE	9	0	9	7	7	0.10
					L. straminellus	SNE	2	0	2	2	2	0
				Macrolepiota	*M. fuliginosa*	E	5	0	5	5	5	0.02
					M. olivascens	E	0	0	0	0	0	-
					M. permixta	E	2	0	2	2	2	0
					M. procera	E	53	2	55	51	51	0.03
					M. venenata	T	0	0	0	0	0	-
			Amanitaceae	Amanita	*A. aminoaliphatica*	T	3	0	3	3	3	0
					A. caesarea	E	0	0	0	0	0	-
					A. citrina	T	40	7	47	28	28	0.07
					A. decipiens	M	0	0	0	0	0	-
					A. echinocephala	SNE	0	0	0	0	0	-
					A. excelsa	SNE	3	0	3	1	1	-
					A. franchetii	SNE	7	0	7	7	7	0.02
					A. gemmata	T	29	3	32	27	24	0.02
					A. muscaria	T	254	9	263	237	235	0.02
					A. ovoidea	T	24	0	24	24	24	0.02
					A. pantherina	T	70	4	74	60	57	0.03
					A. phalloides	M	65	3	68	55	53	0
					A. proxima	T	15	0	15	15	15	0
					A. rubescens	E	69	8	77	67	66	0.03
					A. strobiliformis	E	4	0	4	3	3	0.01
					A. verna	M	6	1	7	3	2	0
					A. virosa	M	39	6	45	37	37	0,03
			Cortinariaceae	Cortinarius	*C. cedretorum*	NE	9	0	9	8	8	0.03
					C. orellanus	M	3	1	4	4	4	0
					C. purpurascens	NE	35	11	46	37	37	0.01
					C. semisanguifluus	M	0	0	0	0	0	-
					C. cystidiatus	E	5	10	15	15	15	0.01

			Entolomateceae	Clitopilus	*C. prunulus*	E	22	20	42	38	28	0.08
				Entoloma	*E. clypeatum*	E	23	7	30	26	23	0.01
					E. lividoalbum	SNE	1	3	4	4	3	0
					E. rhodopolium	SNE	41	13	54	54	54	0.12
					E. sepium	E	7	0	7	5	5	0
					E. sinuatum	T	12	5	17	14	7	0.02
			Inocybaceae	Inocybe	*I. bongardii*	T	8	3	11	7	7	0.03
					I. cookei	T	10	0	10	9	8	0.68
					I. geophylla	T	49	5	54	43	42	0.34
					I. maculata	T	38	1	39	31	21	0.25
					I. rimosa	T	42	4	46	34	33	0.12
			Lyophyllaceae	Calocybe	*C. gambosa*	E	3	2	5	5	5	0
				Lyophyllum	*L. decastes*	E	40	5	45	43	43	0.05
			Omphalotaceae	Gymnopus	*G. aquosus*	E	20	0	20	18	18	0.02
					G. brassicolens	NE	1	0	1	1	1	-
					G. confluens	NE	66	3	69	67	63	0.02
					G. dryophilus	E	97	6	103	93	90	0.09
					G. erythropus	NE	14	1	15	13	13	0
					G. fusipes	T	6	4	10	10	10	0
					G. hariolorum	NE	0	1	1	1	1	-
					G. impudicus	NE	5	1	6	6	6	0.08
					G. ocior	NE	22	2	24	24	24	0.02
					G. peronatus	NE	16	2	18	18	18	0.01
				Omphalotus	*O. olearius*	T	22	0	22	19	19	0.01
				Rhodocollybia	*R. butyracea*	NE	36	7	43	34	34	0.01
					R. prolixa	NE	1	1	2	2	2	0.17
			Psathyrellaceae	Lacrymaria	*L. lacrymabunda*	NE	11	4	15	12	11	0.05
				Panaeolus	*P. papilionaceus*	T	17	3	20	16	16	0.01
				Psathyrella	*P. candolleana*	T	135	27	162	146	146	0.09
				Parasola	*P. conopilea*	T	17	1	18	14	14	0.01
			Physalacriaceae	Armillaria	*A. cepistipes*	E	107	2	109	101	101	0.01
					A. gallica	E	353	2	355	340	339	0.01
					A. mellea	E	194	0	194	172	162	0.06
				Desarmillaria	*D. tabescens*	E	12	0	12	0	0	-
			Strophariaceae	Agrocybe	*A. molesta*	E	0	0	0	0	0	-
					A. praecox	E	18	7	25	23	23	0.02
				Cyclocybe	*C. cylindracea*	E	11	0	11	9	9	0.06
				Galerina	*G. marginata*	M	87	15	102	82	82	0.01
					G. uncialis	SNE	0	0	0	0	0	-
					H. laterinum	NE	9	2	11	9	9	0

				Hebeloma	*H. sinapizans*	T	59	2	61	60	60	0
				Hypholoma	*H. capnoides*	E	31	11	42	38	37	0.13
					H. fasciculare	T	95	7	102	77	75	0.06
					H. lateritium	T	3	5	8	8	8	0
				Pholiota	*P. mutabilis*	E	3	0	3	3	3	0
				Psilocybe	*P. semilanceata*	T	20	4	24	19	19	0
			Tricholomataceae	Clitocybe	*C. candicans*	T	1	2	3	3	3	0
					C. dealbata	T	11	1	12	9	6	0.01
					C. nebularis	T	13	3	16	16	16	0.01
					C. phaeophthalma	T	10	0	10	9	9	0.01
					C. phyllophila	T	11	6	17	17	17	0.01
					C. rivulosa	T	7	0	7	5	5	0.05
				Clitopaxillus	*C. alexandri*	SNE	12	0	12	11	11	0
				Infundibulicybe	*I. geotropa*	E	1	3	4	4	4	0
					I. gibba	E	51	3	54	52	52	0.02
					I. mediterranea	E	1	0	1	1	1	-
				Lepista	*L. glaucocana*	E	1	0	1	1	1	-
					L. irina	E	24	5	29	24	24	0.04
					L. nuda	E	64	6	70	67	66	0.04
					L. panaeola	E	14	0	14	13	13	0
					L. personata	E	1	0	1	1	1	-
					L. sordida	NE	55	6	61	55	48	0.01
				Leucopaxillus	*L. gentianeus*	NE	2	0	2	2	2	0
					L. tricolor	E	9	1	10	7	1	-
				Tricholoma	*T. argyraceum*	E	52	7	59	54	54	0.05
					T. atrosquamosum	E	3	3	6	4	4	0.02
					T. basirubens	E	0	0	0	0	0	-
					T. bresadolanum	T	0	0	0	0	0	-
					T. bufonium	T	2	0	2	2	2	0.12
					T. equestre	T	34	2	36	31	27	0.02
					T. filamentosum	E	2	7	9	9	9	0
					T. fracticum	T	1	0	1	1	1	-
					T. gausapatum	E	0	0	0	0	0	-
					T. joachimii	SNE	3	3	6	4	4	0.06
					T. quercicola	SNE	0	0	0	0	0	-
					T. saponaceum	T	49	16	65	54	51	0.02
					T. scalpturatum	E	64	1	65	63	62	0.02
					T. sciodes	T	3	0	3	3	3	0.01
					T. sejunctum	T	18	2	20	19	13	0.09
					T. sulphureum	T	28	5	33	28	28	0.08
					T. terreum	E	65	5	70	65	60	0.03
					T. triste	E	4	0	4	3	3	0.01
					T. virgatum	E	8	5	13	13	13	0.01
		Auriculariales	Auriculariaceae	Auricularia	*A. auricula-judae*	E	189	1	190	161	159	0.04
					B. aereus	E	35	0	35	30	30	0.01

		Boletales	Boletaceae	Boletus	*B. edulis*	E	238	7	245	214	212	0.03
					B. pinophilus	E	64	4	68	55	55	0
					B. reticulatus	E	46	0	46	31	31	0.01
				Butyriboletus	*B. fechtneri*	E	18	0	18	17	16	0
					B. pseudoregius	E	16	0	16	15	11	0.01
					B. regius	E	12	0	12	10	8	0
				Caloboletus	*C. calopus*	SNE	4	2	6	5	5	0
					C. radicans	SNE	4	0	4	3	3	0.05
				Imperator	*I. luteocupreus*	T	5	0	5	5	5	0
					I. rhodopurpureus	T	10	0	10	8	8	0
				Neoboletus	*N. erythropus*	E	11	0	11	10	10	0.03
					N. luridiformis	E	7	3	10	7	7	0.02
				Rubroboletus	*R. lupinus*	T	6	0	6	5	5	0
					R. rhodoxanthus	T	6	0	6	6	6	0.02
					R. satanas	T	6	0	6	6	6	0.03
				Suillellus	*S. comptus*	E	7	0	7	6	5	0
					S. luridus	E	35	2	37	34	34	0.02
					S. pulchrotinctus	T	4	0	4	4	3	0
					S. queletii	E	6	0	6	6	6	0.05
				Suillus	*S. bellinii*	E	11	0	11	11	11	0
					S. bovinus	E	109	4	113	107	106	0.01
					S. cavipes	E	23	0	23	22	18	0.02
					S. collinitus	E	33	0	33	27	27	0.02
					S. granulatus	E	72	2	74	68	66	0.05
					S. grevillei	E	47	5	52	46	46	0.02
					S. luteus	E	112	5	117	103	99	0.01
					S. mediterraneensis	E	5	0	5	2	2	0.03
					S. viscidus	E	31	0	31	29	29	0.01
				Tylopilus	*T. felleus*	SNE	31	1	32	28	28	0.10
			Gyroporaceae	Gyroporus	*G. castaneus*	T	18	2	20	11	11	0.02
					G. cyanescens	E	13	1	14	9	9	0.16
			Hygrophoropsidaceae	Hygrophoropsis	*H. aurantiaca*	SNE	22	5	27	23	23	0.08
			Paxillaceae	Paxillus	*P. involutus*	M	148	7	155	113	113	0.04
					P. rubicundulus	T	6	0	6	4	4	0
			Rhizopogonaceae	Rhizopogon	*R. luteolus*	SNE	10	1	11	9	8	0.02
					R. roseolus	SNE	120	0	120	103	103	0.07
			Sclerodermataceae	Scleroderma	*S. citrinum*	T	49	2	51	36	33	0.08
					S. polyrhizum	T	16	0	16	13	7	0.06
					S. verrucosum	T	25	0	25	23	21	0.10
		Cantharellales	Cantharellaceae	Cantharellus	*C. alborufescens*	E	21	0	21	1	1	-
					C. amethysteus	E	15	2	17	11	11	0.02
					C. cibarius	E	48	3	51	17	16	0.15
					C. cinereus	E	3	0	3	3	3	0
					C. cornucopioides	E	0	0	0	0	0	-
					C. ferruginascens	E	37	0	37	0	0	-
					C. ianthinoxanthus	E	0	0	0	0	0	-
					C. lutescens	E	4	5	9	8	8	0.04

				Craterellus	*C. tubaeformis*	E	28	6	34	27	27	0.10
				Pseudocraterellus	*P. undulatus*	E	0	1	1	1	1	-
		Gomphales	Gomphaceae	Ramaria	*R. aurea*	E	3	0	3	0	0	-
					R. botrytis	E	16	0	16	16	15	0.13
					R. flava	E	8	0	8	8	8	0.03
					R. flavescens	E	12	0	12	10	10	0
					R. flavosalmonicolor	E	2	0	2	1	1	-
					R. formosa	T	11	0	11	9	7	0.04
					R. pallida	T	3	0	3	3	3	0
					R. sanguinea	E	3	0	3	3	3	0
					R. subbotrytis	SNE	7	0	7	6	6	0.07
		Russulales	Russulaceae	Lactarius	*L. blennius*	T	13	3	16	7	7	0.01
					L. chrysorrheus	NE	35	4	39	33	33	0.03
					L. controversus	NE	14	0	14	12	12	0.01
					L. decipiens	NE	12	0	12	10	10	0.01
					L. deliciosus	E	224	6	230	205	196	0.01
					L. mediterraneensis	NE	7	0	7	7	7	0
					L. pallidus	NE	6	0	6	6	6	0
					L. pyrogalus	NE	3	3	6	4	4	0.01
					L. quieticolor	NE	26	7	33	24	24	0
					L. quietus	NE	28	4	32	27	27	0.01
					L. scrobiculatus	T	19	1	20	17	14	0.01
					L. tesquorum	NE	5	0	5	5	5	0
					L. torminosus	T	31	11	42	38	34	0.02
					L. vellereus	T	15	1	16	14	14	0.02
					L. zonarius	T	8	0	8	7	6	0.04
				Russula	*R. caerulea*	SNE	5	3	8	6	6	0.01
					R. chloroides	NE	19	10	29	23	22	0.03
					R. cyanoxantha	E	56	7	63	48	48	0.12
					R. delica	NE	26	2	28	23	18	0.04
					R. foetens	T	27	4	31	26	25	0.15
					R. grata	SNE	15	4	19	17	17	0.01
					R. heterophylla	E	9	0	9	4	4	0.05
					R. illota	T	12	1	13	12	12	0
					R. ionochlora	E	42	2	44	41	41	0
					R. maculata	NE	13	1	14	10	9	0.02
					R. mustelina	E	13	4	17	5	5	0
					R. nobilis	NE	0	1	1	1	1	-
					R. olivacea	E	10	0	10	8	8	0.06
					R. parazurea	E	29	2	31	10	8	0
					R. persicina	NE	12	0	12	9	7	0.02
					R. queletii	T	47	2	49	17	17	0.03
					R. sanguinea	NE	17	3	20	14	10	0.03
					R. sardonia	NE	7	3	10	9	9	0
					R. subfoetens	NE	5	1	6	5	5	0.03
					R. torulosa	T	1	1	2	2	1	-

					R. turci	SNE	18	5	23	21	21	0.03
					R. vesca	E	34	5	39	33	33	0.01
					R. vinosobrunnea	E	0	0	0	0	0	-
					R. virescens	E	32	0	32	25	23	0.04
Ascomycota	Pezizomycetes	Pezizales	Discinaceae	Gyromitra	*G. esculenta*	M	30	0	30	26	23	0.03
					G. gigas	M	27	1	28	27	27	0
					G. infula	M	43	3	46	44	43	0.01
			Morchellaceae	Morchella	*M. elata*	E	54	0	54	54	53	0.04
					M. esculenta	E	114	2	116	97	97	0.14
					M. semilibera	E	35	0	35	35	35	0
				Verpa	*V. bohemica*	T	12	1	13	13	13	0.07
					V. conica	E	9	0	9	9	9	0.11
					V. digitaliformis	SNE	1	0	1	1	1	-
					TOTAL		6715	555	7270	6183	5977	

Table S2. Classification of the genera from Basidiomycota based on the ITS species identification performance reported by Badotti et al. [28] and comparison with target genera and species from this study.

ITS identification performance	genera	Number of target genera	Number of target species
Good	*Agaricus* spp., *Amanita* spp., *Auricularia* spp., *Chlorophyllum* spp., *Entoloma* spp., *Gymnopus* spp., *Leucoagaricus* spp., *Lepiota* spp., *Lepista* spp., *Leucopaxillus* spp., *Lyophyllum* spp., *Macrolepiota* spp., *Parasola* spp., *Psathyrella* spp., *Psilocybe* spp., *Rhodocollybia* spp., *Russula* spp., *Scleroderma* spp., *Suillus* spp.	19 (33.9%)	110 (47.2%)
Intermediate	*Armillaria* spp., *Boletus* spp., *Cortinarius* spp., *Hebeloma* spp., *Hypholoma* spp., *Inocybe* spp., *Paxillus* spp., *Ramaria* spp., *Rhizopogon* spp., *Tricholoma* spp.	10 (17.8%)	53 (22.7%)
Poor	*Butryboletus* spp., *Lactarius* spp.	2 (3.6%)	18 (7.7%)
Not evaluated	*Caloboletus* spp. *Clitocybe, Craterellus* spp. *Galerina* spp., *Gyroporus* spp., *Leucocoprinus, Omphalotus, Panaeolus, Pholiota Tylopilus* spp.<br>*Agrocybe* spp., *Calocybe* spp., *Cantharellus* spp., *Clitopaxillus* spp., *Clitopilus* spp., *Cyclocybe* spp, *Desarmillaria* spp., *Hygrophoropsis* spp. *Imperator* spp., *Infundibulicybe* spp., *Lacrymaria* spp,	23 (41.1%)	52 (22.3%)

	Neoboletus spp., *Pseudocraterellus* spp., *Rubroboletus* spp., *Suillellus* spp		
